# Artisanal and Small Gold Mining and Petroleum Production as Potential Sources of Heavy Metal Contamination in Ecuador: A Call to Action

**DOI:** 10.3390/ijerph18062794

**Published:** 2021-03-10

**Authors:** José Luis Rivera-Parra, Bernardo Beate, Ximena Diaz, María Belén Ochoa

**Affiliations:** 1 Departamento de Petróleos, Escuela Politécnica Nacional, Ladrón de Guevara E11-253, 170525 Quito, Ecuador; mabe_ochoa@hotmail.com; 2 Departamento de Biología, Escuela Politécnica Nacional, Ladrón de Guevara E11-253, 170525 Quito, Ecuador; 3 Departamento de Geología, Escuela Politécnica Nacional, Ladrón de Guevara E11-253, 170525 Quito, Ecuador; bernardo.beate@epn.edu.ec; 4 Departamento de Metalurgia Extractiva, Escuela Politécnica Nacional, Ladrón de Guevara E11-253, 170525 Quito, Ecuador; ximena.diaz@epn.edu.ec

**Keywords:** artisanal and small gold mining, contaminant movement, Ecuador, environmental risks, extractive industries

## Abstract

Mining and petroleum production are the source of many elements and base materials fundamental for our modern way of life. The flip side of these keystone industries is the environmental degradation they can cause if not properly managed. Metallic mining and petroleum production can contaminate the local ecosystem with sediments, chemicals used in the industrial processes and heavy metals, part of the metallic ore or oil reservoir. The objective of this project was to analyze the spatial distribution of the presence of different potentially hazardous elements that make up the metallic deposits and oil reservoirs in Ecuador, focused mainly on artisanal and small-scale gold mining (ASGM) districts. Additionally, we were interested in analyzing this information under the local political and administrative contexts which are key to determining how likely it is that mismanagement of the local mineral deposits and petroleum exploitation projects will end up causing environmental degradation. An extensive and intensive literature search was conducted for information on the presence and concentration of 19 potentially harmful elements. We analyzed data on 11 metallic deposits throughout Ecuador and a major oilfield in the Ecuadorian Amazon basin. We used geographic information systems to analyze the spatial distribution of these reservoirs and their mineral compositions. The results indicated a widespread distribution and high concentration of elements potentially harmful for human health, such as mercury, cadmium and arsenic, throughout the metallic deposits in Ecuador. This is particularly true for long-exploited ASGM districts, such as Ponce-Enríquez, Portovelo-Zaruma and Nambija. This study highlights the importance of understanding geological diversity and its potential risks to better protect the biological diversity and public health of its inhabitants. Furthermore, we consider our work not as a call to stop ASGM mining nor petroleum production, but on the contrary as a strong call to plan every mining and petroleum production project considering these risks. Moreover, our work is a call to action by the local government and authorities to stop corruption and fulfill their duties overseeing the activities of mining and petroleum companies, stopping illegal mining, helping ASGM communities to improve their environmental standards, finding alternative income sources and protecting the local environment.

## 1. Introduction

Mining and petroleum production provide the basic resources that sustain a diverse array of industries which are keystones of our modern way of life. Precious metals such as gold or platinum are fundamental for electronics [1] and even for cutting edge medical therapies such as gene editing [2]. The same applies for the products derived from petroleum and the petrochemical industry, which create ubiquitous plastics and base materials for countless industries. The flip side of mining and petroleum production lies in the environmental impacts they can generate, such as water contamination, people displacement and large-scale disasters such as tailings dam breakage or large oil spills, e.g., [3,4,5,6,7,8,9]. Cases such as the Brumadinho dam collapse, which occurred in 2019, are extreme examples of the impacts large-scale mining can cause [10]. In the petroleum industry, oil spills such as Exxon Valdez [11] or Deepwater Horizon [12] are well-known cases of major environmental disasters which affected kilometers of coastline and countless species at sea. Even when examples like these are the ones that come to mind when thinking about environmental impacts from mining and petroleum production, usually, the environmental impacts are more discrete. Usually, small-scale impacts contaminate local rivers and ravines that are later used by local communities and contaminate local ecosystems. These impacts have two main origins: problems handling the different chemicals and petrous material products of mining and petroleum production [13], and risks directly related to the chemistry of the exploited deposits and reservoirs and its surrounding rock, including the formation water that lies in the oil reservoirs [14].

The geochemistry of the mineral deposit itself and its associated surrounding formation can be the primary predictor of potential environmental contamination [15]. Metallic deposits besides gold, silver or copper have other metals that can have the potential to affect human health [16]. Is not uncommon that gold deposits are rich in arsenic or cadmium, two elements with extensive evidence of toxicity to human beings [17]. Oil reservoirs as well can have different geochemical compositions, particularly in its formation water. Produced water or formation water is usually rich in salt and can have heavy metals such as cadmium or lithium in its composition [18]. Thus, understanding the spatial distribution and relative concentration of potentially toxic elements in precious metal and oil deposits is paramount for predicting potential risks when mining the ore or producing petroleum. Furthermore, this information should be a keystone for developing adequate environmental management plans.

Another natural factor, beyond human control, relevant to understanding the environmental risks of different mining operations is the amount of rain (precipitation) a site receives [19]. Once the rocks are extracted from the underground, the chemical composition and state of oxidation can release to the environment potentially harmful heavy metals and produce acid rock drainage [20]. Therefore, areas with high precipitation or humidity have an increased risk of acid rock drainage and mobilization of heavy metals from the reservoir rock or surrounding sterile material, in the case where there is no proper management of tailing ponds and dumps.

Heavy metals such as cadmium, lead or mercury are found in the produced water fraction of the overall petroleum production [18]. Water, as the universal solvent, in the case where it is spilled, will carry all the chemicals and metals that are dissolved and readily transport them, infiltrating the soil and contaminating nearby water bodies [21]. In this case, local precipitation and other local ecosystem characteristics such as soil cover, topography and soil composition determine the likely destiny of any heavy metals that escape the petroleum facilities. Oil spills are the most visible environmental impacts, but formation water spills or mishandling can reach further and have longer term impacts. Produced water, due to its salt content, can directly and immediately affect plants and fresh water organisms. The heavy metal content of produced water can persist in the environment and enter the food chain of the local human population and the local ecosystem [22]. In the case of the petroleum industry, we focused our analysis on a single major oilfield in the Ecuadorian Amazon basin, the Auca oilfield. This particular oilfield is responsible for 18% of Ecuadorian oil production and produces an average of 468,000 daily barrels of produced water per day.

Mining, particularly artisanal and small-scale gold mining (ASGM) also has a special heavy metal contamination risk associated with the chemical handling needed for the ore processing. This is particularly true for gold mining, where extensive Hg is used to extract gold through an amalgamation process. Mercury is a known teratogen and carcinogenic, and if effluents are not properly treated, these chemicals end up in rivers and ravines and through bio-accumulation enter the food chain affecting the health of the local community [23,24]. The use of mercury for gold amalgamation has been prohibited in Ecuador, but the reality is that its use is still widespread [25].

Ecuador has several very rich metallic deposits. Some have been exploited for many decades or even centuries by ASGM. Well-known ASGM areas in Ecuador are Nambija, Zaruma-Portovelo and Ponce-Enríquez [26,27,28,29,30,31]. Other major deposits have been recently discovered and due to their characteristics need more industrialized exploitation techniques. Deposits such as Mirador or Fruta del Norte are in the early steps of producing copper and gold, respectively, and are being managed by large multinational mining companies.

The geological and mineralogical richness of Ecuador can be explained by the diversity of geological environments and the geological processes involved, such as volcanism, which is the origin of mineral deposits [32]. Thus, most mineral deposits are located in the slopes of the Andes.

Bioaccumulation of heavy metals through food chains is a very well-documented process, where heavy metals such as Hg and Cd enter the environment [33]. Once exposed to sun, humidity and other effects, they transform into bioavailable chemical species that can be absorbed by living organisms. In each step of the food chain their absolute concentration increases, until they reach a point where they can have toxic effects, either to animals themselves or to human beings that consume contaminated products, such as fish from rivers or crops grown on contaminated soil or irrigated with contaminated water [33]. Toxicity effects of heavy metals depend on the chemical compound, element concentration, exposure route and time. Acute or chronic effects can vary from dermal lesions, neurological problems to different type of cancers [34,35,36,37,38,39,40,41,42,43,44,45,46,47]. Drinking water is one of the major recognized sources for chronic effects of heavy metals [48,49].

A key piece of information for land use planning and risk analysis from these extractive industries is to know the contamination’s potential and its spatial distribution. Understanding the risks associated with the natural composition of soil, rocks and underground geological formations allows local authorities to better plan the distribution of productive activities in their territories [50]. Moreover, it also helps to establish adequate environmental guidelines and better practice requirements for local industries. This is particularly relevant for ASGM communities, that usually are in need of technical training to improve safety and environmental standards and have a suite of associated social problems that need to be addressed by the local authorities.

Thus, the objective of this study was to determine the most likely composition and potential distribution of heavy metal contamination in various metallic deposits, mainly from ASGM districts, throughout Ecuador and a major oilfield in the Ecuadorian Amazon basin. More specifically, the interest was in: (1) analyzing the specific composition of the deposits that contain precious metals in the major ASGM areas in Ecuador; (2) the spatial distributions of the different deposits and reservoirs and their variation when analyzed element by element; (3) determination of the presence and distribution of heavy metals and metalloids with potential to harm living beings in some of the major mineral deposits and reservoirs of oil fields in Ecuador; and (4) analyzing this information under the local political and administrative contexts, which are key to determining how likely it is that mismanagement of the local mines and petroleum exploitation projects will end up causing environmental degradation.

Ecuador has several large deposits of precious metals and oil reservoirs [51]. Even though there is interest from the government in developing the mining industry, there is resistance from the local communities because of fear of contamination of their water sources and agricultural lands [52]. This concern comes in part due to previous experience with Ecuadorian oil production that started in the 1960s, which has a history of environmental impacts [53]. This paper attempts to determine the potential distribution of heavy metal contamination in different habitats due to these large-impact industries. This information may be key for better environmental planning, better informed community positions and even better community relationship programs. We decided to include oil production in the analysis because this industry represents a major source of income for the Ecuadorian government and is an interesting contrast between an obligated industrialized, regulated and legal activity, and ASGM which lacks regulations, control and law enforcement. It is performed by individuals or small companies instead of major national or international companies.

## 2. Materials and Methods

### 2.1. Study Areas

**Figure 1:**
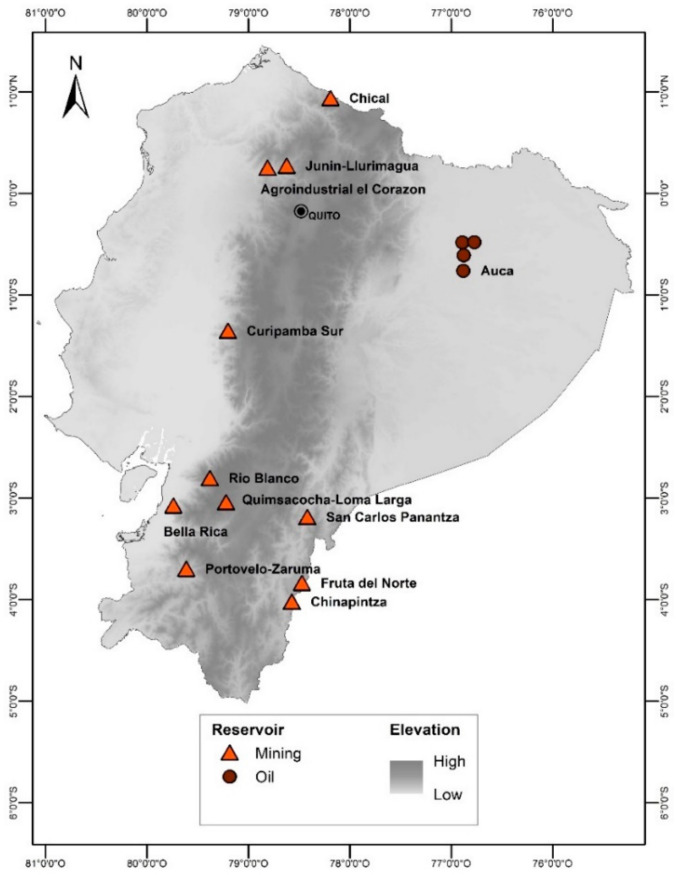
Map of study area. Location of the studied metallic reservoirs and studied oil field.

We analyzed 11 mineral deposits in Ecuador; some are new prospective medium- to large-scale mining projects and others are long-term artisanal and small-scale mining (ASGM) areas. The studied areas of medium- to large-scale mining projects were: Chical, Junin-Llurimagua, Agroindustrial el Corazón, Fruta del Norte, San Carlos Panantza, Quimsacocha-Loma Larga, Río Blanco and Curipamba sur (Figure 1). On the ASGM areas, we analyzed the mineral reservoirs of Chinapintza, Bella Rica and Portovelo-Zaruma (Figure 1). These reservoirs have a long history of exploitation and a known track record of environmental problems [26,27,28,29,30,31]. In the case of the petroleum industry, we analyzed samples from 4 different points located throughout the Auca oilfield (Figure 1).

### 2.2. Data Collection

The collected data come from different sources including published research papers as primary sources, technical reports from government repositories, such as environmental impact assessments approved by the Ecuadorian Ministry of Environment, and online databases. The intent was to find specific concentrations of the different elements in the deposits, but in several cases, qualitative presence/absence information was found. Table 1 summarizes the sources where the information was obtained. An extensive and intensive search of information was conducted and compiled in a database that included information from a total of 19 elements (Table 2).

### 2.3. Spatial Distribution

To better represent the distribution of the different elements, the deposits and reservoirs were georeferenced for each mining area and points within the Auca oilfield, and projected using ArcGIS ver 10.7 [71], using a WGS 84 coordinate system. Maps for each element in the analysis were developed, classifying the concentration in relative ranges, and also comparing the concentration with the World Health Organization recommendations for water consumption. We chose each element to be studied by analyzing and classifying the elements and their risk according to Williams et al. [72]. We decided to compare the information of concentration of the different elements in the deposit with the WHO drinking water standards [73] only as a proxy for safety. We decided to perform this comparison with the logic that if the rocks were once mined from the deposit and the precious metals extracted, the leftovers or residues will be left in tailing dams or dumps. Therefore, these elements in contact with water might mobilize and end up in the local hydric system. The WHO standards were used as a general reference [73] for the safety of the elements themselves and the safety threshold for concentration. However, processes like oxidation, bioavailability of the element and bioaugmentation need to be further explored to assess in detail the potential effects and risks of heavy metal contamination in different areas. In the case of produced water, it was considered that there is a chance of spills and also if the water is injected it might end up in superficial aquifers and the superficial hydric system, due to cracks in the well’s cement seal or connectivity between underground formations. Furthermore, the specific management of the different mining areas can be the main factor to determine if there will be major environmental impacts or not.

Table 2 summarizes the known information regarding the effects of the studied elements and their potential for bioaccumulation. This information is fundamental to understanding the potential risks the local environment may face.

**Table 2:** Potential health effects of the studied elements, maximum recommended concentration by the WHO [73] and potential for bioaccumulation.

Component	Maximum Recommended Concentration [mg/L] (WHO, 2017)	Health Effects (WHO, 2017)	Bioaccumulation Potential
Aluminum [Al]	0.1–0.2	Acceleration Alzheimer’s disease onset	Medium [34]
Arsenic [As]	0.01	Dermal lesions (hyperpigmentation and hypopigmentation), peripheral neuropathy, skin, bladder and lung cancers and peripheral vascular disease	High [35]
Cadmium [Cd]	0.003	Kidney cancer	High [36,37]
Chromium [Cr(VI)]	0.05	Lung cancer, other types of cancer	Medium [37,38]
Cobalt [Co]	-----	Lung diseases and respiratory effects (Kim et al., 2006)	Medium [37]
Copper [Cu]	2	Gastrointestinal bleeding, hematuria, intravascular hemolysis, methaemoglobinaemia, hepatocellular toxicity, acute renal failure and oliguria	High [39]
Fluoride [F^-^]	1.5	Skeletal fluorosis	Medium [40]
Iron [Fe]	0.3	Turbidity, color and bad taste to water	Low [41]
Lead	0.01	Neurodevelopmental effects, cardiovascular diseases, impaired renal function, hypertension, impaired fertility and adverse pregnancy outcomes	High [42]
Manganese [Mn]	0.1–0.5	Adverse neurological effects (WHO, 2004)	Medium [38]
Mercury [Hg]	0.006	Severe disruption of any tissue with which it comes into contact in sufficient concentration, neurological and renal disturbances	High [43]
Molybdenum	0.07	None	Low [44]
Nickel [Ni]	0.02	Carcinogenic	Medium [59]
Selenium [Se]	0.04	Gastrointestinal disturbances, discoloration of skin, decayed teeth, hair and nail loss	Medium [37]
Silver [Ag]	0.1	Argyria	Medium [37]
Vanadium [V]	----	Lung diseases and respiratory effects	Medium [37]
Zinc	3	Pulmonary distress and gastroenteritis	Medium [39]
Antimony [Sb]	0.02	Gastrointestinal mucosa irritated, abdominal cramps, diarrhea and cardiac toxicity	High [46]
Tin [Sn]	------	Acute gastric irritation	High [47]

## 3. Results and Discussion

### 3.1. General Description of Studied Metallic Reservoirs

Information from 11 metallic deposits in Ecuador was found. This will be analyzed case by case for the main mineralogical and geochemical composition.

#### 3.1.1. Agroindustrial El Corazón

This deposit is found surrounded by andesitic lava and tuff. The most abundant precious metal is gold, which is located in siliceous hydrothermal breccia with quartz veins. There are known anomalies of iron, mercury and copper in this deposit and it is rich in sulphur. Moreover, silver is also prevalent in this deposit.

This deposit also has very high concentrations of arsenic, chrome, copper, lead, mercury, molybdenum, nickel, silver, vanadium, zinc and antimony. Moreover, the most common mineral species are: cassiterite {SnO_2_}, wolframite {CaSiO_3_-Ca_3_}, molybdenite {MoS_2_}, sphalerite {ZnS}, chalcopyrite {CuFeS_2_}, magnetite {Fe^2+^Fe^3+^_2_O_4_}, argentite {Ag_2_S}, enargite {Cu_3_AsS_4_}, galena {PbS}, pyrite {FeS_2_}, marcasite {FeS_2_} [54,55,56].

#### 3.1.2. Bella Rica

The rocks in the Bella Rica are surrounded mainly by volcanic rocks, andesite, basalt, breccia and diabase. Free gold is common in the deposit, as well as multiphase veins of gold associated with quartz and carbonates. The deposit is quite rich in sulphide and arsenic.

The most common minerals found in the deposit are: pyrrhotite (Fe(_1−x)_S), arsenopyrite {FeAsS}, chalcopyrite {CuFeS_2_}, epidote {Ca_2_Fe^3+^Al_2_O(OH)}, wurtsite {ZnS}, galena {PbS}, hematite {Fe_2_O_3_}, molybdenite {MoS_2_}, magnetite {Fe^2+^Fe^3+^_2_O_4_}, cuprite {Cu_2_O}, chalcocite {Cu_2_S}, covellite {CuS}, malachite {Cu_2_CO_3_(OH)_2_} [57].

#### 3.1.3. Chical

Volcanic rocks surround the deposit. The deposit has quartz veins in the shape of roots embedded in the volcanic rocks. There are evident deposits of precious metals in the quartz veins, with sulphide, zinc and copper evident as well.

The most common minerals found in this reservoir are chlorite, epidote {Ca_2_Fe^3+^Al_2_O(OH)}, calcite {CaCO_3_} and traces of pyrite {FeS_2_}. Specifically, in the quartz veins there is up to 2% of pyrite, up to 1% chalcopyrite and less than 1% of galena and sphalerite [58].

#### 3.1.4. Chinapintza

The mineralization occurs within an intrusive felsic volcanic complex from the late Cretaceous period. The deposit is mostly formed by epithermal deposition on quartz veins. It is very rich in sulphur associated with metals, mainly: pyrite, sphalerite, galena, arsenopyrite, pyrrhotite, chalcopyrite, bornite, tetrahedrite and malachite [59].

#### 3.1.5. Curipamba Sur

It is located within an important hydrothermal alteration zone. It includes massive sulphur deposits. The precious metals are associated with sulphurs. The most common mineral species are: tennantite {Cu_12_As_4_S_13_}, pyrite {FeS_2_}, tetrahedrite {(Cu,Fe)_12_Sb_4_S_13_}, galena {PbS}, chalcopyrite {CuFeS_2_}, sphalerite {ZnS}, covellite {CuS}, bornite {Cu_5_Fe S_4_}, azurite {Cu_3_(CO_3_)_2_(OH)_2_}, arsenopyrite {FeAsS} [60].

#### 3.1.6. Fruta del Norte

The deposit is surrounded by andesites from the Misahualli formation and feldspar porphyry intrusions. The mineralization is characterized by quartz-sulphide veins, particularly in the central and north of the deposit, where also gold and silver are more abundant. The main minerals found are: orthoclase {KAlSi_3_O_8_}, rodocrosite {MnCO_3_}, barite {BaSO_4_}, marcasite {FeS_2_}, pyrite {FeS_2_}, sphalerite {ZnS}, galena {PbS}, chalcopyrite {CuFeS_2_}, alabandite {MnS}, stibnite {SbS_3_}, arsenopyrite {FeAsS}, acanthite {Ag_2_S}, freibergite {Ag_6_Cu_4_Fe_2_Sb_4_S_13_}, boulangerite {Pb_5_Sb_4_S_11_}, jamesonite {Pb_4_FeSb_6_S_14_}, valentinite {Sb_2_O_3_}, senarmontite {Sb_2_O_3_} [61].

#### 3.1.7. Junin-Llurimagua

The desposit is located with intrusive granodiorite bodies. The mineralization is associated with bornite {Cu_5_FeS_4_}, chalcopyrite {CuFeS_2_} and molibdenite {MoS_2_} [62,63].

#### 3.1.8. Portovelo-Zaruma

The surrounding rock is formed by andesites and tuff. There are three mineralization phases, one mainly formed by quartz-pyrite-chlorite-hematite, one a quartz-pyrite-chalcopyrite and one a polymetallic quartz rich in galena and sphalerite and galena-chalcopyrite. The most common minerals are: bornite {Cu_5_FeS_4_}, hematite {Fe_2_O_3_}, tennantite {Cu_12_As_4_S_13_}, tetrahedrite {(Cu,Fe)_12_Sb_4_S_13_}, magnetite {Fe^2+^Fe^3+^_2_O_4_}, molibdenite {MoS_2_}, argentite {Ag_2_S}, freibergite {Ag_6_Cu_4_Fe_2_Sb_4_S_13_}, nagyagite {(Pb(Pb,Sb)S_2_)((Au,Te))}, proustite {Ag_3_AsS_3_}, bournonite {PbCuSbS_3_}, pyrite {FeS_2_}, chalcopyrite {CuFeS_2_} [64].

#### 3.1.9. Quimsacocha-Loma Larga

The deposit is located in andesitic lava flows, with breccias and tuff. The mineralization is rich in sulphur. Thus, the most common minerals are: pyrite {FeS_2_}, enstatite {MgSiO_3_}, lizardite {Mg_3_Si_2_O_5_(OH)_4_}, chalcopyrite {CuFeS_2_}, covelite {CuS}, luzonite {Cu_3_AsS_4_}, tennantite {Cu_12_As_4_S_13_}, tetrahedrite {(Cu,Fe)_12_Sb_4_S_13_} [65].

#### 3.1.10. San Carlos Panantza

The deposit is found surrounded by granite and leucogranite. The most common minerals are: chalcopyrite {CuFeS_2_}, molibdenite {MoS_2_}, magnetite {Fe^2+^Fe^3+^_2_O_4_}, pyrite {FeS_2_}, anhidrite {CaSO_4_}, gypsum {CaSO_4_·2H_2_O}, chalcocite {Cu_2_S}, malachite {Cu_2_CO_3_(OH)_2_}, chrysocolla {(Cu,Al)_4_H_4_(OH)_8_Si_4_O_10_·nH_2_O)}, cuprite {Cu_2_O} [66,67].

#### 3.1.11. Río Blanco

The surrounding rock is mainly andesite feldspar, tuff and breccia with volcanic sandstone and dacitic tuff. The gold–silver mineralization is found in veins several hundred meters long. The most common minerals are: electrum {Au,Ag}, pyrite {FeS_2_}, pyrrhotite {Fe_1−x_ S}, pyrargyrite {Ag_3_SbS_3_}, tetrahedrite {(Cu,Fe)_12_Sb_4_S_13_}, arsenopyrite {(Cu,Fe)_12_Sb_4_S_13_}, sphalerite {ZnS}, galena {PbS}, chalcopyrite {PbS} [70].

#### 3.1.12. Nambija

The host rock is a complex sequence of tuff, lapilli tuff with skarn and pyroclastic breccia. The main mineralization in the Nambija deposit is gold, with some porphyry associations of Cu-Au and Cu-Mo. The gold is usually free with a 90% purity and 7–10% silver. The most common minerals are: pyrite {FeS_2_}, chalcopyrite {CuFeS_2_}, pirrhotite {Fe_(1−x)_S}, sphalerite {ZnS} and galena {PbS} [68,69].

### 3.2. Distribution of Anomalies of Element Concentration

Table 3 summarizes the concentration distribution of the studied elements. Moreover, the comparison of the element concentration with WHO standards are shown for each element.

Several anomalies were found distributed throughout Ecuador. Any value significantly higher than the other reported values is considered an anomaly. Some remarkable findings are as follows.

In the case of aluminum, it was present in Fruta del Norte in southeastern Ecuador and in the Auca oilfield in high levels. In these same places it exceeds the WHO recommendations. On the other hand, arsenic was present throughout Ecuador. The concentrations were particularly high in Quimsacocha and Agroindustrial Corazón. It exceeds the WHO limit in Quimsacocha, Bella Rica, Fruta del Norte, Agroindustrial el Corazón and Junin-Llurimagua. The fact that the values were very high in Quimsacocha is very worrisome considering that the area is a source of drinking water for Cuenca, one of the largest cities in Ecuador. This is even more concerning when analyzing the mercury concentrations (Table 3). The highest concentration of natural mercury among the analyzed sites was found at Quimsacocha. The Auca oilfield and all the northwestern sites showed presence of natural mercury in levels above the WHO threshold, and other sites had mercury presence as well. Mercury is known to bioaccumulate once it has been methylated. Therefore, these findings suggest that there might be a significant presence of mercury in the local food chains due to natural geochemical factors.

Another element known to bioaccumulate is cadmium. This element was present in high levels in the Auca oilfield and Junin-Llurimagua. Chical showed lower concentrations, but still exceeded the WHO limits. The Auca oilfield is located in the Ecuadorian Amazon basin, where there are numerous cocoa plantations. The contamination with cadmium, a product of produced water spills, has already started affecting cocoa bean exports because the product exceeded the maximum Cd levels allowed by the European Union [74]. To the best of our knowledge, there is no information on the effects on human beings in that area, but it is likely that they are consuming contaminated crops.

In the case of lead, it was found in very high concentrations in most sites. This is probably related to the abundance of galena in the metallic deposits. This is another element that can cause major metabolic disruption in the human body and is widely distributed in Ecuador. Chromium is present and particularly prevalent in the northern sites (Table 3). It exceeds the WHO limits in Chical, Agroinsutrial Corazón and Junin-Llurimagua. Cobalt exceeds the safety limits in two points in Auca and Chical, Agroindustrial Corazón, Junin-Llurimagua and Fruta del Norte (Table 3). Ecuador’s metallic deposits tend to be very rich in copper. Therefore, in most sites the concentration is very high. The results are similar for nickel and silver (Table 3), found in most of the sites in high concentrations.

We found references of the presence of manganese only for Agroindustrial el Corazón and Auca (Table 3). However, there are reports of manganese in groundwater wells near the Bella Rica deposit [75]. In the case of fluoride, we only found information for the Auca oilfield, where it was above the WHO threshold.

Other elements such as selenium, molybdenum, vanadium and zinc were found in different sites in Ecuador with locally high values (Table 3). We found no information regarding the presence of francium, radium or actinium.

### 3.3. A Call to Action

Our analysis shows the widespread and prevalent presence of heavy metals throughout Ecuador. This indicates the potential for the local environment to suffer from heavy metal contamination and environmental degradation. However, this is only a risk which has not materialized in most of the studied deposits.

An important point to make is that the development of this project was hindered by the lack of published information about the geochemistry of most of the different mineral deposits and oil fields. It was particularly difficult to find published information for specific mineral deposits traditionally or currently exploited by artisanal and small-scale gold mining (ASGM), such as Chinapintza, Portovelo-Zaruma or Bella Ric, probably, because these types of mining operations do not have a formal geological exploration. A special case is the mining deposit of Nambija, located in the Ecuadorian Amazon basin and considered one of the richest gold deposits in Ecuador. Even though this deposit has been exploited for more than 50 years by artisanal gold miners, there was no information on its geochemistry available or published. It is very likely that there is significant geochemical information on these and other deposits, but that information is not widely available. Thus, studies such as this are hindered and our ability to give information for better land use planning and even improve community relations that would benefit the different mining and petroleum companies, is limited. To us, this is a call to strengthen the field of geochemistry in Ecuador and motivate the publication of the information by companies and governmental agencies.

Most of the studied areas are known deposits but there are no active mining activities to exploit them. Ecuador is interested in developing its deposits in a regulated and industrialized way. Thus, the largest deposits, such as Fruta del Norte, Mirador or Quimsacocha are either in the initial producing steps or in licensing phases to large national or international companies [76]. Thus, we still have time to avoid the environmental problems that are common in small and artisanal gold mining areas, that have been operating for more than 50 years.

Artisanal areas such as Zaruma-Portovelo, Ponce-Enríquez, Chinapintza and Nambija have a well-documented and sad record of environmental and social problems [26,27,28,29,30,31]. These very rich gold deposits have been exploited in a completely unregulated manner with no government intervention to stop or at least regulate these activities, likely due to corruption networks with interests in these activities protecting and extorting the miners [77,78].

An extreme case of illegal mining in Ecuador happened in another reservoir (not included in our analysis due to lack of information) called Buenos Aires, located near Junín-Llurimagua. In the last two years the Buenos Aires deposit became known to artisanal miners from other parts of Ecuador and in the course of one year, more than 8000 people colonized this mountainous area to extract gold illegally. Finally, in early 2020, the government evicted the illegal miners, but the environmental damage, mainly due to deforestation, was already there. Nobody knows how much gold was extracted, but it is common knowledge that the material rich in gold was transported to southern Ecuador, 500 km away, to Ponce-Enríquez and Portovelo where benefit plants operate. Thus, it is very likely that a corruption network developed to protect those shipments from police controls [79,80].

In the case of oil, the exploration and production is performed by national or international companies under a strict legal framework. In the past, the regulatory framework was very lax and there are still environmental passives (mainly old oil spills) across north eastern Ecuador. Even though nowadays the oil industry in Ecuador is improving its environmental track record, there are still oil spills and produced water spills [81]. As part of the current legal framework, the companies have the obligation to report any oil or produced water spills. In reality, it is likely this is not happening, due to the limited on-the-ground control and corruption.

A positive recent piece of news is that Ecuador joined in October 2020 the Extractive Industries Transparency Initiative (EITI) [82]. This is one of a few steps the government is taking to strengthen its institutions and prevent corruption. Valuable commodities such as gold, silver, or oil can provide the resources to underpin the economy of a developing country, such as Ecuador, but this is not possible if most of the resources are lost due to corruption [83].

Thus, the responsibility lies in the central Ecuadorian government, responsible for licensing, and in the local governments and Environmental Ministry offices, responsible for monitoring and assessing the fulfillment of the legal framework and environmental action plans. There is an urgent need to stop corruption and to strengthen the local institutions so they have the resources and, most importantly, the will to protect the local environment. Therefore, our work, besides showing the spatial distribution of these potential risks, it is a call to action to all the local stakeholders interested in exploiting these valuable resources and in protecting the local environment. It is also a call to the Ecuadorian academia, to reach out to local stakeholders, miners, farmers and environment ministry officers to collaborate on finding technical solutions to exploit these resources in a safer and sustainable manner.

To the best of our knowledge, this work is the first attempt to understand the bigger risk the natural composition of metallic deposits represents in Ecuador. Furthermore, we consider our work not as a call to stop mining, but on the contrary, this is a strong call to plan every mining and petroleum production project considering these risks. Moreover, we want to motivate the empowerment of the local municipalities to control their territories and protect their environment. It is also a call to the local municipalities to develop, update and enforce land use plans in their jurisdictions. Ecuador is a biologically rich and diverse country, and is also incredibly diverse and rich in its geological resources. We need to consider both sources of richness for future planning and development.

## 4. Conclusions

The major ASGM districts in Ecuador, Bella Rica, Portovelo-Zaruma, Nambija and Chinapintza, have an extensive presence of heavy metals in their deposits. Thus, the lack of formal environmental planning, control and law enforcement as well as technical mining training are major hazards for the local environment and population.

There is a need to further study the ecosystems surrounding the mineral deposits to confirm the presence of heavy metal contamination and understand if the specific local conditions are in fact allowing contaminant movement.

Ecuador is embarking on the large-scale development and exploitation of its metallic deposits. Thus, we still have time to prevent further environmental damage if the government officers and institutions fulfill their duty to oversee the operation of legal, small and large mining operations, stop illegal mining and monitor the operation of oil companies.

## Figures and Tables

**Table 1:** Sources of information for the different metallic reservoirs and location coordinates (WGS84).

Reservoir Name	Latitude	Longitude	Main Metal	Current State	Reference
Agroindustrial el Corazón	0.249	−78.811	gold, copper, silver	ASGM	[54,55,56]
Bella Rica	3.076	−74.741	gold, silver	ASGM	[57]
Chical	0.935	−78.190	gold, copper, silver	ASGM	[58]
Chinapintza	−4.022	−78.572	gold	ASGM	[59]
Curipamba-sur	−1.356	−79.202	gold	Exploration for large scale mining	[60]
Fruta del Norte	−3.836	−78.471	gold, silver	Early large scale mining	[61]
Junin-Llurimagua	0.269	−78.623	copper, gold	Exploration for large scale mining	[62,63]
Portovelo-Zaruma	−3.698	−79.612	gold, silver	ASGM	[64]
Quimsacocha-Loma larga	−3.043	−79.220	gold	Exploration for large scale mining	[65]
San Carlos Panantza	−3.189	−78.419	copper, gold	Exploration for large scale mining	[66,67]
Nambija	0.103	−78.789	gold	ASGM	[68,69]
Rio Blanco	−2.806	−79.366	gold	Early medium scale mining	[70]

**Table 3:** Summary of concentrations of the studied elements in the different deposits. Present-NC indicates that qualitative information of presence was found, but no details of specific concentration. An * indicates the concentration exceeds the WHO [73] safety limits.

	Agroindustrial el Corazón		Auca	Bella Rica	Chical	Chinapintza	Curipamba-Sur
Aluminum [ppm]	Present-NC		0.86 *	-	0.1	-	-
Arsenic [ppm]	290 *		0.01	0.48 *	-	-	Present-NC
Cadmium [ppm]	Present-NC		0.13 *	-	0.02 *	-	-
Chromium [ppm]	207 *		0.05	-	10 *	-	-
Cobalt [ppm]	17 *		0.55 *	-	11 *	-	-
Cooper [ppm]	570 *		0.01	21 *	0.06	Present-NC	19,200 *
Fluoride [ppm]	-		9 *	-	-	-	-
Iron [ppm]	Present-NC		105 *	-	-	-	Present-NC
Lead [ppm]	12 *		0.006	-	17 *	16,800 *	3700 *
Manganese [ppm]	430 *		2.9 *	-	-	-	-
Mercury [ppm]	30 *		0.00003	-	0.1 *	-	-
Molybdenum [ppm]	5.44 *		0.06	-	0.1 *	-	-
Nickel [ppm]	25 *		0.64 *	-	0.05 *	-	-
Selenium [ppm]	-		0.01	-	0.005	-	-
Silver [ppm]	1.5 *		-	-	-	Present-NC	58 *
Vanadium [ppm]	24 *		0.7 *	-	8 *	-	-
Zinc [ppm]	18 *		0.85	-	0.2	30,200 *	35,200 *
Antimony [ppm]	21 *		-	-	-	-	-
Tin [ppm]	-		-	-	-	-	-
	**Fruta del Norte**	**Junin-Llurimagua**	**Nambija**	**Portovelo-Zaruma**	**Quimsacocha-Loma larga**	**Rio Blanco**	**San Carlos Panantza**
Aluminum [ppm]	9400 *	-	-	-	Present-NC	-	-
Arsenic [ppm]	300 *	1.1 *	-	-	2200 *	Present-NC	-
Cadmium [ppm]	-	0.1 *	-	Present-NC	Present-NC	-	-
Chromium [ppm]	-	22 *	-	-	Present-NC	-	-
Cobalt [ppm]	10 *	5.5 *	-	Present-NC	Present-NC	-	-
Cooper [ppm]	-	377 *	Present-NC	40,000 *	5900 *	Present-NC	6400 *
Fluoride [ppm]	-	-	Present-NC	-	-	-	-
Iron [ppm]	10,300 *	-	Present-NC	Present-NC	32 *	-	16,800 *
Lead [ppm]	Present-NC	3.2 *	Present-NC	40,000 *	Present-NC	-	-
Manganese [ppm]	-	-	-	-	Present-NC	-	-
Mercury [ppm]	0.03 *	0.1 *	-	Present-NC	50 *	Present-NC	-
Molybdenum [ppm]	-	2.4 *	Present-NC	-	Present-NC	Present-NC	80 *
Nickel [ppm]	10 *	9 *	-	Present-NC	Present-NC	-	-
Selenium [ppm]	-	1 *	-	Present-NC	Present-NC	-	-
Silver [ppm]	12 *	Present-NC	Present-NC	Present-NC	47 *	Present-NC	1.3 *
Vanadium [ppm]	-	94 *	-	-	-	-	-
Zinc [ppm]	0.007	63 *	Present-NC	100,000 *	182 *	Present-NC	-
Antimony [ppm]	-	-	-	Present-NC	-	Present-NC	-
Tin [ppm]	-	Present-NC	Present-NC	-	-	-	-

## Data Availability

The data presented in this study are available on request from the corresponding author.
